# An Address Delivered to the Graduating Class of the Atlanta Medical College. February 28th, 1884

**Published:** 1884-04

**Authors:** Wm. H. Felton

**Affiliations:** Cartersville, Ga.


THE ATLANTA
MEDICAL AND SURGICAL JOURNAL.

VOL. I. APRIL, 1884. No. 2.

### Original Communications.

### AN ADDRESS DELIVERED TO THE GRADUATING CLASS OF THE ATLANTA MEDICAL COLLEGE, FEB. 28TH, 1884.

BY HON. WM H. FELTON, M. D., CARTERSVILLE, GA.

*Gentlemen of the Class and Respected Audience:*

"Physicians mend or end us,
Secundem artem—but although we sneer
In health—when sick we call them to attend us
Without the least propensity to jeer."—*Byron.*

The "beloved physician" is the scriptural appellation of a man who had distinguished himself in the profession which especially attracts our attention at this time.

It is a term more expressive, because more comprehensive, than the descriptive word "honored."

This latter word simply means dignity, rank, title—that which is illustrious—but the former descriptive term includes all these; "dignity and conspicuous eminence," conjoined with such tender regard for others as to awaken our affections—our love. It is a higher grade of honor, having its throne in the heart. The intellect may be dazzled with the brilliant powers of the illustrious man—both intellect and heart do homage to the "beloved physician."

We are not surprised that in the earlier ages of the human family, such men were esteemed as superhuman, and were honored and worshiped as deities.

In Greece, Æsculapius had his temples, and those who served his altars were the learned Asclepiadæ, who ministered to the sick and diseased multitude.

Hippocrates, the "father of medicine," was the remote descendant of the Asclepiadæ, through a long line of physicians, and, although he greatly improved their theories and their mode of practice by the study of Anatomy and the symptoms of disease, he was esteemed as deriving his "healing art" from divine sources. Even in the celebrated Alexandria School of Medicine, which was founded by that most distinguished surgeon and physician—Herophilus—"priest and physician" were interchangeable descriptive terms. He lived only about three hundred years before the Christian era. All through the ages of Celsus and Galen in Italy, France, and all over Europe, until the fifteenth and seventeenth centuries, inclusive, this learned profession was associated with religious orders—was an appendage to the priesthood. The sacerdotal robe was considered an essential to the successful physician. Legendary traditions, fabulous religious observances and superstitious rites, were all, more or less, relied upon for the cure of disease—though some learned men had during this time made their appearance in the "healing art"—men whose names will live forever because of their anatomical discoveries and their intelligent investigations of the causes and treatment of disease. But the "saving of a patient" was even then considered to be a supernatural work, and connected the physicians in popular opinion with the supernatural world.

About this time—a time when the intellect of the world seems to have experienced a "new birth"—when the world started afresh in its growth—when the genius of invention and discovery gave intimation of its future triumphs; about this time the medical profession, in common with other "callings and occupations," emerged from the clouds into the sunshine.

This was the age that gave to the world Columbus, Luther, Richelieu—Gutenberg and Faust, with their printing press; Newton, Shakespeare, Harvey and Bacon. How suggestive their names! They seem to be the "first fruits" of the revival of learning. The world appears to have been lingering in obscuration under the shadows of the "dark ages," when Providence furnished these men as "motive power" to start the world in that career of glory—of material and physical blessings, of which we are but gathering the early morning flowers.

To Lord Bacon, especially, is science more indebted than to all others.

Not because of discoveries or inventions, for he made neither; but because he gave to the world the great truth—the "philosopher's key," which had already partially unlocked—was then, to some extent, unlocking, and has since most triumphantly thrown open the doors of nature and permitted the multitude to look upon its secrets. This "key" of knowledge was embodied in this thought: "Examine carefully minute and particular facts for the establishment of general laws that we may thereby promote the material and physical welfare of men." That is, master nature by induction that human life may be prolonged and made comfortable; that is, investigate with only the useful in view; that is, advance in knowledge that the conditions of men may be ameliorated.

These were the great thoughts of Bacon. This was the stimulant he applied to the philosophy and science of the world. How it quickened the intellectual pulse of Europe! How superstition and empiricism retired at its touch!

The desire to discover new truths was intensified because the hope of such discovery was inspired by new motives and objects—namely, the physical and material happiness of men. It was no longer "philosophic repose" that men sought after in philosophy. It was no longer "intellectual culture" that men labored to attain by intellectual pursuits. It was no longer the reward of "conscious knowledge" which induced the chemist, physiologist and anatomist to investigate, examine, dissect and experiment. The object henceforth was the prolongation and the betterment of human life.

Macaulay, in speaking of this Baconian system, illustrates it very forcibly with this simple medical suggestion: "The philosophers and scholars of the world before Bacon's time would have looked on a case of small-pox and soliloquized thus: 'There is no evil in small-pox. in disease or in decay.' They produce resignation, detach the mind from all cares and prepare us for the 'inevitable.' But after Bacon's *novum organum*, the 'new instrument,' the 'new method,' these learned men as they looked upon a case of small-pox would have taken out their lancets and commenced vaccinating." This simple illustration solves the Baconian system. This imperial intellect attracted the learning of the world from the unattainable, and directed its labors toward the useful and practical purposes of life. Science no longer sought to raise men above the common wants and necessities of their nature, but it labored to supply their wants. Learning no longer sought to make "humanity perfect, but to render our imperfect nature comfortable."

How this "new method" has advanced the medical profession! How it has carried medical science to the front ranks of the world's learning!

It is said that so late as 1674, French comedians satirized this profession in the play-houses of Paris, and for months the entire city received certain plays, which were caricatures of this profession, with roars of laughter. To lampoon a doctor was considered eminently just and proper. It was deservedly the object at which every jester might safely aim his keenest blade of sarcasm.

But these clouds are gone, and we assert confidently that for work already accomplished, for learning, for power with men, and for universal beneficence, it stands out the foremost "calling and occupation" known to our civilization.

*The Medical profession has for its purpose and object the prolongation and comfort of human life.*

In all ages of the world this has been regarded the highest motive which can prompt human action. There is nothing so valuble to the individual as life. Deprivation of life is the highest penalty of criminal law. Human life in action is the wealth, the power and glory of nations. Governments confer medals and valuable rewards upon men and women who distinguish themselves by saving human life. The prime object of all governments, and of all wise legislation, is the protection and comfort of human life. This is the last analysis of all authorized human effort.

What is the object and purpose in saving human life? It is "the vestibule to another life." It is the preparatory chamber of the unseen and the permanent. Let us save life that the opportunity for usefulness may be prolonged—the space for reformation lengthened—the bread-winners of the household continued in position—the benediction of womanhood protracted—the flowers and fragrance of the domicil preserved. Every life, however debased or exalted, is the sunbeam of some heart—of some home—occupies some field of labor or of suffering—fills some niche for instruction, for warning, or for reproof. Yes, let us save human lives. Are they morally and physically lepers? "Go shew yourselves to the priests!" and "as they went they were healed." Is that life the prop and pillar upon which gentleness and virtue lean for bread and guidance? "If thou hadst been here my brother had not died." Is the "wife's mother sick of a fever," and unable to extend the courtesies and services of the household to the loved and honored guest? "He touched her hand and the fever left her."

Is the son born to wealth and power, "at the point of death," and to whom the father, hoping yet to transmit the honors and emoluments of his family, pleads, "Sir, come down ere my child die?" "Go thy way, thy son liveth;" and at the same hour, yonder in the distant Capernaum, "the fever left him."

Is it congenital blindness?

He anoints the eyes, bids the man wash, and "he came seeing."

To cure—to heal—to save human life, and to make that life comfortable, was the great work of the Great Physician.

Some days ago the legislature of a Western State, with a gallantry and wisdom worthy of all commendation, voted a considerable sum of money and a medal to a young girl who had breasted storm and darkness to give notice to an approaching passenger train of impending disaster; thus by her courage and womanly devotion—risking her own life for the life of others—she succeeded in saving a great number of persons who otherwise would have perished.

Within the last few weeks the National House of Representatives unanimously voted the thanks of the American people to a sailor who had, by personal exertion, saved the lives of two passengers of the ill-fated ship, "City of Columbus." This was a well-deserved and handsomely bestowed tribute. There can be no greater appreciation of merit than the expressed thanks of the American people through their national representatives in Congress. I repeat, it was most appropriate. Perhaps two homes, away somewhere in city or country, were made glad by the heroism of the brave sailor. Perchance, two fathers and mothers—maybe a dozen persons—somewhere on this beautiful earth, will a while longer wear the sunshine in their hearts.

Let us for a moment summon to our presence the name and memory of Edward Jenner. The pupil of John Hunter, receiving his diploma in 1792, and in 1796, making his first successful inoculation of cow-pox, as a preventive of small-pox—a discovery based upon observations he had made twenty years before, for, being the son of a country clergyman, he had grown up among the dairy farms of his native county, Gloucester. He had communicated these impressions at the time they were taking shape in his mind to his personal friend and preceptor, John Hunter. Hunter replied, "*Sir, continue your observations.*"

Here was a young man who had probably never read Bacon's

"*Novum Organum*," but who had caught the spirit of inquiry—which that book had awakened throughout Europe—and who was unconsciously practicing its great lessons, namely, making minute investigations of particular facts for the establishment of general laws looking to the preservation of human life. In obedience to Hunter's suggestion, he "continued his observations," and now as the result of those continued observations, ask the civilized world how many lives have been saved by Edward Jenner?

From its first appearance in Europe in 710 until 1798, this deadly pestilence, small-pox, was the scourge of Europe, from twenty to fifty per cent. of the cases proving fatal, city and country, prince and peasant, being alike its victims. Now the electric bolt, guided by the magicians of science, is scarcely less harmful than this enemy of human life.

Let us briefly, while speaking of Jenner, summon to our presence the name and memory of his great preceptor, John Hunter, the founder of modern surgery. A Scotchman by birth, an Englishman by residence, imperfectly educated in his early life, and yet as a benefactor to mankind, as a substantial laborer in the fields of knowledge having for their object the prolongation of human life, he probably stands out in history as having no equal preceding him and no superior after him.

It is asserted that in prosecuting his studies in anatomy and pathology, he dissected at least five hundred different species of animals. At his death his museum contained ten thousand preparations, illustrative of human and comparative anatomy, physiology, pathology and natural history. His discoveries and suggestions were numerous and invaluable.

He discovered the muscularity of the arteries, the muscularity of the iris, that the red globules of the blood are formed after the other component parts of the blood are formed, and that their function is to minister to the strength rather than the repair of the system. He first recognized and explained inflammation of the veins, and many other anatomical and physiological truths. His great field of research, however, was pathology, disease, perverted structures and functions. He absolutely labored to build up and establish a science of the abnormal—and he succeeded—the abnormal in the animal and vegetable world, in organic and inorganic matter.

He saved life. Take one discovery illustrative of this fact.

He first suggested that aneurism might be cured by tying the artery at a distance from the seat of disease. Before his day, the treatment was to cut down into the sac of the aneurism and tie the artery above and below the incision, a dangerous and often fatal operation.

One of Hunter's distinguished eulogists says that this one suggestion has "saved thousands of lives." It is saving life to-day, and will continue to save lives for the coming centuries.

Take one other illustration of Hunter's efficiency in saving human life:

It is said by one of his distinguished biographers that "never had physiology been so incorporated with surgery, never been so applied to investigation of disease and the suggestion of treatment, as it was by this master workman. And to him we owe it, that for "the last half century the foundations of English surgery have been changing from a basis of empiricism to a basis of science."

Another eulogist of Hunter says: "Those who have traced modern surgery to its true source will not fail to see, in the principles which Hunter established, the germs of all the improvements which have since been introduced."

Now estimate, if you can, the number of human lives annually saved and made comfortable by the vast number of learned and skillful surgeons throughout the civilized world—on battle-fields, in armies, in hospitals, on ship-board, in private practice. Tell me the number of the rescued, and I will approximate the number of lives annually saved by the broad-shouldered, rough-visaged and somewhat uncouth Scotchman, John Hunter.

No branch of the profession has improved more rapidly than *surgery*. For the last one hundred years its growth has been wonderful, and its triumphs unparalleled in the history of science. What an array of names in the Larreys, the Dupuytrens, the Velpeaus, the Coopers, the Abernathys, the Bells, the Listons, the Simpsons, the Guthries, and, crossing the waters to this country, the Physicks, the Warrens, the Gibsons, the Motts, the Dudleys and McDowells of the world. America has no cause to blush in this array of mighty names. It is said that Valentine Mott, dead only a few years, "tied more arteries and saved more human lives, by simply tying of arteries, than any man who ever lived." I mention not the living in this list of names, because my eulogies might offend the modesty of some living Georgians. But take the mighty host, and their colleagues and successors, as they move with cautery, with ligature, with bandage, with couching-needle, with anesthetic, and with knife, amid the diseases and casualties of the world, and tell me how many are annually saved and made comfortable by the learning and skill of this "innumerable company" of surgeons throughout the world?

It is said that when Hippocrates died all the world united with the citizens of Argos in erecting to his memory a statue of solid gold, commemorative of his skill and professional worth But this "innumerable company" of beloved physicians and surgeons have built, and are erecting for themselves, monuments in human hearts, "more to be desired than gold, yea than much fine gold."

If the saving of two shipwrecked persons can command an expression of thanks from the American Congress, then let us for one moment call to our presence the memory of that "Board of Physicians" to whom the Royal Society of London referred the question of the great plague, popularly termed the Black Death, in 1665. This contagious fever entered Europe in the year 544. From that time until 1665, it is estimated that twenty-five millions of human lives in Europe alone were destroyed by this pestilence—a pestilence which literally "walked in darkness," and "a destruction" which literally "wasted at noonday." It is said the fatality of the disease blocked the progress of civilization for several centuries.

This particular epidemic of 1665, John Evelyn says, "carried to the grave in the city of London sixty-eight thousand persons." In obedience to that law of suffering mentioned, I believe, by Shakespeare, "When sorrows come they come not single spies, but in battalions."

The succeeding year the great fire of London swept over four hundred and thirty-six acres in the heart of the city, consuming some fourteen thousand houses. In less than two years the city loses nearly one-half of its population and two-thirds of its buildings—one by pestilence, the other by fire. John Evelyn winds up his description of both calamities with these words: "London was, but is not."

At the same time the moral and political atmosphere of London was charged with poisons almost as deadly as those which were sweeping its population to the grave. The Protestant was charging the Papist with being the incendiary of London. The Papist was making counter-allegations. The surviving adherents of Cromwell vehemently denounced the Court as having offended Heaven by its licentiousness, and as having brought on the pestilence as a scourge from God. The Monarchists were with equal zeal alleging it was a divine punishment for the unrighteous execution of the late King.

The reigning King, Charles the Second, and his Court, the most abandoned in sensuality known to the annals of Europe, were daily oppressing the people and extorting money to be expended upon royal favorites. What a spectacle to men and angels did London at this time present! Charles the Second on the throne, Jeffreys on the bench, Oates and Dangerfield the prominent figures in the picture! Political and religious factions prosecuting their deadly feuds! The block red every day with the blood of some Sidney, or some Russell, the apostles of liberty!

Not a door-post in all London whose sprinkled lintels could shut out the "Angel of Death."

It was a time when pestilence and treason, when bigotry and blood, when tyranny and sensuality, held high carnival over the smoking embers of the world's metropolis.

But that was the opportunity of great minds. Times of general calamity and confusion have ever been productive of the greatest intellects. "The purest ore is produced from the hottest furnace. The brightest thunderbolt is elicited from the darkest storm"

In all this confusion this Board of Physicians, under the direction and patronage of the Royal Society of London, then in its infancy, continued its labors. These learned men were not attached to any faction; they recognized no sectarian or political creed; they were consecrated to their profession and humanity. Unmindful of the surrounding confusion, not afraid when a thousand were falling at their side and ten thousand at their right hand, they "continued their observations." They examined every fact and minute particular connected with the pestilence, looking to the establishment of some general law for its extirpation.

Finally they report. Substantially, that report is as follows: Cause—*atmospheric insalubrity, produced by wet, marshy soils;* general neglect of *cleanliness on the part of domestic and municipal authorities.* Remedy, preventive—"*Thorough drainage.*" It was the first intimation the world had received as to the cause of the plague. When the cause of disease is discovered, it is well-nigh subdued, requiring only the removal of that cause. The fire had opened a broad field for the engineers, and placing themselves under the direction of these medical architects of the coming London, perhaps the most perfect system of sewerage in the world was then commenced. The city authorities enforced hygienic regulations with a severity at that time unparalleled. The continent caught up the idea as an inspiration from God. Drainage as a preventive remedy for plague!

It was simple. It was only the falling apple, from which was evolved the laws of gravity. It was only the lid of the tea-kettle, which gave motive power for the machinery of the world. It was only the muscular action of the frog's leg, which sent messages in a moment around the globe. How simple! how grand!

Drainage as a preventive of plague!

The result was, that with the exception of a few sporadic cases, and occasionally an unimportant local epidemic, this "Black Death" has been "stamped out" from the face of Europe.

But some one may say that for this exemption from disease the world is more indebted to civil engineers and civil authorities than to the medical profession. It may be said that these authorities have enforced a strict observance of domestic and public hygiene. Whence this word hygiene? Preserving health and preventing disease. Hygieia, the daughter of Æsculapius, was the goddess by whom the ancient Asclepiadæ swore when entering upon the duties of their profession; and I assert that every idea from that day until the present, valuable to the world for the prevention of disease, has been originated by the medical profession. Civil engineers and civil authorities have only been the executive forces of this imperial power behind the throne. In preserving health and preventing disease, the medical profession has accomplished more than in the "art of healing." The wisdom and beneficen e of its labors are seen in the gradual and decided increase of the mean duration of human life.

This mean duration of life increases as new and wider fields of knowledge open to the profession. It is estimated that this average of human life has increased twenty-five per cent. in the last fifty years. Fifty years ago the mean duration of life in France was twenty-eight years. Now it is thirty-four years. At Geneva, in the seventeenth century, the average of human life was fourteen years. Now it is forty-five years. In London, and all the larger cities of England, in the seventeenth century, one in every twenty of the inhabitants annually died. Now, one in every forty. These cheering figures, showing the prolongation of life, are only strikingly marked in those countries where the science of medicine has made the most decided progress. Not that this profession anticipates a reversal of the decree, which allots to man three-score and ten years; but it may anticipate from evidence already furnished—that with its continued exploration of every field of knowledge—with its untiring investigation of every pathological development and the subserviency with which every newly discovered truth in science advances this high calling of men, that the day approaches when the average duration of human life, under the guidance and supervision of this profession, will closely approximate the limitations assigned it by its Divine Author and benefactor. All trustworthy records refute the old cynicism, that men are degenerating physically. "It has been conclusively shown that we are larger; that we are stronger; that we are longer-lived; that we resist disease better, and that we are better looking than our ancestors. This wonderful change is not attributable to a change of type in diseases, but the change of type is in the art of healing and preventing disease."

This profession, medicine, suggested and established quarantine in the fourteenth century as a prevention for disease. It is true, the germ of quarantine existed in the time of Moses, but it was indefinite and uncertain. It only assumed practical shape during the fourteenth century. This hygienic agency, under the supervision of the medical profession, has improved in efficiency continually; and has, especially during the present century, been established on common-sense principles. Let us shut out all exotic diseases. Let us bar out every germ of contagion, and when they enter or become epidemic, let us "stamp them out" by sanitary science. Let us stamp them out by destroying the food which sustains the pestilence. Sanitary science and sanitary work were almost a nullity in the world until the Act of Parliament in 1834, establishing a "medical commission" to supervise the "poor law" of England. From that medical commission has grown some of the grandest results which have ever crowned the medical profession. From this beginning boards of health, sanitary commissions, sanitary legislation and all the invaluable agencies and appliances which control disease and lengthen human life have expanded. Under these hygienic agencies, the percentage of deaths among children in the larger cities of the world has been strikingly diminished. The demand of this age upon the medical profession is to "*save the children.*" This demand would be more fully met if the profession could reach and control the prolific sources of disease, which are found in the larger commercial centres of the world—their squalid poverty, their idleness, their vices, their gross ignorance, their crowded tenements, their defective drainage, and their insufficient and improper supplies of food and water.

These larger cities of the world are a sort of *fomites*, which carry contagion and death upon every passing breeze, and sweep to untimely graves the young and beautiful.

Much remains, however, to be accomplished. To the reproach of human knowledge and the disgrace of civil authorities, a majority of our race die young. Infancy and youth, beauty and strength, are yet the sheaves gathered by the Great Reaper. Yet we constantly hear from the battle-fields, where physicians and death are each struggling for the mastery, some shout which tells of newly-won victories and gives assurance of future triumphs.

We are prepared to assert and maintain that each of these future triumphs of the medical profession will be, as in the past, the product of progressive knowledge in science, and a clearer and truer insight into the nature of that living body with which it has to deal.

If this be true, then the first essential qualification of this profession is *learning*—familiarity with all the sciences—mental, moral and natural science. Know all things—especially be masters of all that we call nature, for nature is only man and his relative surroundings—for man is the objective point of all divine and human work.

The age in which we live demands technical and universal learning in this profession.

> "Hear him reason in divinity
> And, all admiring, with an inward wish
> You would desire the king were made a prelate.
> List his discourse of war, and you shall hear
> A fearful battle render'd you in music;
> Turn him to any cause of policy,
> The Gordian knot of it he will unloose,
> Familiar as his garter."

All the branches of human learning, of philosophy and of science, are, like the different members of the body, "fitly joined together." They are mutual supports. They replenish with oil each other's lamps—the light and warmth from which lamps all centre and blaze in one magnificent Argand—*man*.

The day has come when empiricism and quackery must disappear. There are too many railroads, telegraphs, printing presses and electric lights for "quack salving-cheating mountebanks"—longer to survive. Light is everywhere, and the pretenders in medicine are quickly detected and exposed. Some one has defined a quack to be a man "who pours drugs, of which he knows little, into a body of which he knows less;" that is, a man who is uneducated technically—who is unlearned generally—a man who speculates on human life and finds a harvest in credulous ignorance and the necessities of suffering humanity.

But as the darkness is dispelled by approaching light, so the ignorance and illiteracy, upon which quackery has fed and fattened, is retiring before the intellectual progress of the age.

This profession of medicine will be found fully abreast with that intellectual progress. At no time in its history have its members been more active and untiring in their investigation of disease. In Egypt, in India, throughout Asia, in the West Indies, through the crowded cities of the world, wherever pestilence and epidemics, disease and death, are doing their work, there this army of scholars —these veterans of science, with unflinching courage, are "continuing their observations."

Think not, graduates of to-night, that you are entering a field which has been exhausted of its wealth and culled of its richest and most desirable conquests. All that has been conquered in the past is your property to-day. The intellectual treasures of by-gone years are yours. It is humanity's capital invested in the profession, and, after a season, humanity will demand its " own with usury." Happy the profession if it can, then, point to the prevailing diseases of this age as captives at its feet.

